# CRISPR-Cas adaptive immunity and the three Rs

**DOI:** 10.1042/BSR20160297

**Published:** 2017-07-17

**Authors:** Tom Killelea, Edward L. Bolt

**Affiliations:** School of Life Sciences, University of Nottingham, Nottingham, NG7 2UH, UK

**Keywords:** CRISPR, recombination, replication

## Abstract

In this summary, we focus on fundamental biology of Clustered Regularly Interspersed Short Palindromic Repeats (CRISPR)-Cas (CRISPR-associated proteins) adaptive immunity in bacteria. Emphasis is placed on emerging information about functional interplay between Cas proteins and proteins that remodel DNA during homologous recombination (HR), DNA replication or DNA repair. We highlight how replication forks may act as ‘trigger points’ for CRISPR adaptation events, and the potential for cascade-interference complexes to act as precise roadblocks in DNA replication by an invader MGE (mobile genetic element), without the need for DNA double-strand breaks.

## Overview: from CRISPR-Cas biology to genome editing and back to biology

**Figure 1:**
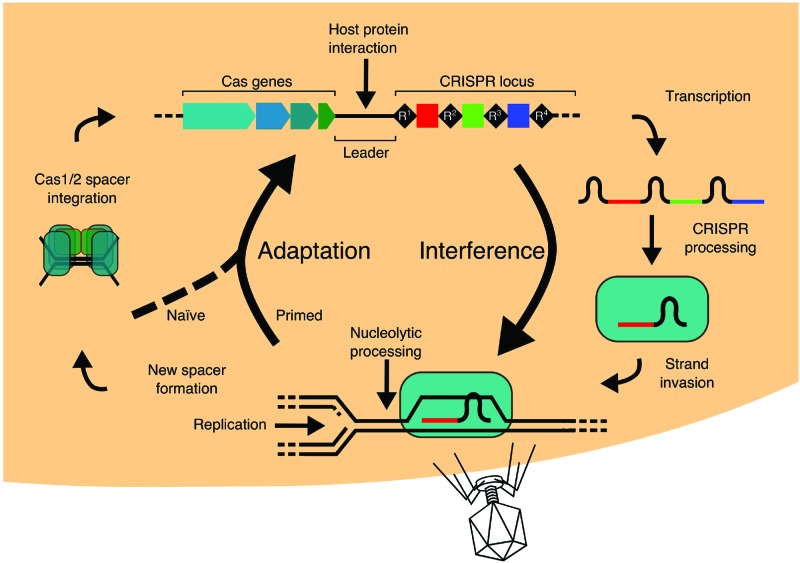
Overview of the stages of CRISPR-Cas adaptive immunity. During ‘adaptation’, DNA fragment ‘protospacers’ from an MGE are captured and integrated into a CRISPR locus, where they become a new spacer coupled to synthesis of a new DNA repeat. Transcription a CRISPR into RNA leads to loading of crRNA fragments into ‘interference’ complexes that are called ‘Cascades’ in many bacteria and archaea. Cascades and analogous interference complexes, catalyse base pairing between crRNA and MGE DNA, leading to further adaptation and nuclease activity directed to the MGE.

CRISPR (Clustered Regularly Interspersed Short Palindromic Repeats)-Cas (CRISPR-associated proteins) systems provide prokaryotes with adaptive immunity against MGEs (mobile genetic elements). Immunity is affected through two major components that interact with other non-Cas host proteins in cellular biology in ways that are still being elucidated: (i) a specialized DNA locus called CRISPR and (ii) *cas* genes, encoding Cas proteins. The overarching mechanisms by which CRISPR-Cas provides defence against MGEs are very similar (Figure 1), throughout a diversity of CRISPR-Cas types and subtypes: (i) MGE DNA fragments (‘protospacers’) are captured by Cas1 enzymes working in concert with Cas2 or analogous proteins, for integration of captured DNA into CRISPR as new ‘spacers’. This process is called spacer acquisition or adaptation, (ii) transcription of CRISPR from a ‘leader’ promoter generates RNA that is processed into ‘crRNA’ (CRISPR RNA), which is the cargo in ribonucleoprotein complexes that target MGE DNA, (iii) base-pairing of crRNA and DNA within the ribonucleoprotein complex forms an ‘R-loop’ (RNA-loop), in which an RNA–DNA duplex is formed, leaving one DNA strand unpaired. In some instances, this can be an intermediate provoking homologous recombination (HR), but in the context of CRISPR-Cas immunity, it is a target for nucleolytic degradation of MGE DNA that may also stimulate further adaptation. R-loop formation and nucleolytic processing of MGE DNA are collectively called ‘interference’. Readers requiring more mechanistic detail of these stages of CRISPR-Cas systems are directed to recent review articles [1,2].

There are two major classes in CRISPR-Cas system [3,4]. In type I systems, interference requires a complex of multiple proteins bound to crRNA, often called as ‘Cascade’ [5], that acts with a nuclease enzyme [6]. Type II systems utilize a single protein interference complex, most famously Cas9 [7]. Each class is further classified into multiple types and subtypes, described in [3-7]. Mechanistic details for the events shown in Figure 1 are detailed below where required, but the interested reader is also guided towards [1,2,8]. The biographical details of how CRISPR-Cas was discovered, including the derivation of CRISPR-associated jargon, and its subsequent development into a tool for biotechnology are detailed in [9-12]. Research into fundamental biology of CRISPR-Cas has been essential to underpin the ‘genome editing revolution’ that is now available in kit-form from retailers, and is widely reported in scientific literature, mainstream media, courtrooms and within some interesting niches of the blogosphere. Discoveries of new naturally occurring CRISPR-Cas interference proteins with differing properties to Cas9, such as Cpf1 and C2c1 [13-18], suggest new markets for novel gene editing tools. Novel chimeric protein fusions of Cas9 are also generating new ways to manipulate nucleic acids [19-21].

In this short survey, we focus on emerging ideas in analysing the fundamental biology of CRISPR-Cas in bacteria and archaea. We focus on interactions of CRISPR-Cas proteins with non-Cas host cell proteins that drive DNA replication, repair and HR, in the context that replication forks recruited for MGE replication, and R-loops formed by CRISPR-Cas interference are trigger points for capturing of MGE DNA for acquisition of new spacers, building CRISPR-based immunity.

## CRISPR-Cas immunity: adaptation and interference

Adaptation (also called ‘spacer acquisition’) drives CRISPR-Cas immunity depositing into a CRISPR locus new spacers from MGE DNA. Spacer crRNA transcripts then provide the means for R-loop-based interference, when crRNA base pairs with the complimentary DNA sequence in an MGE. Physical and functional linkage between adaptation and interference, first identified in *Escherichia coli* [22] is a potent feedback circuit for updating CRISPR-Cas immunity, described later. Adaptation can be subdivided into two major events, target ‘DNA capture’ and its ‘integration’ into a CRISPR locus, extensively reviewed recently in [2]. A ‘Cascade’ ribonucleoprotein complex targets a payload of crRNA to MGE DNA to catalyse CRISPR interference in most bacterial and archaeal type I CRISPR systems. There are significant differences in structural details and protein composition of Cascades from different phyla, but they share an underlying principle of assembling crRNA into the complex for pairing to DNA, forming an R-loop, first elucidated in *E. coli* [23]. Interaction of Cascade with a nuclease, typically a Cas3-family translocase-nuclease, completes interference by degradation of MGE DNA and this may present new substrates for adaptation. Cas9 also forms R-loops for interference reactions [24], but negates the requirment for a Cas3-family translocase-nuclease by possessing intrinsic nuclease activity that creates a DNA double-strand break within the R-loop. This sequence-sensitive R-loop formation and nuclease activity within a single Cas9 protein polypeptide has made it specially suited for genome editing reactions.

## Adaptation: integration of MGE DNA as CRISPR spacers

Two proteins widely distributed across all the CRISPR-Cas systems, Cas1 and Cas2, catalyse the capture and integration of new spacers into CRISPR loci. *E. coli* Cas1–Cas2 so far provides the most complete picture of the molecular events of protospacer integration and, to a lesser extent, protospacer capture. In two crystal structures, Cas1 homodimers are bound on either side of a Cas2 dimer in a butterfly-like (Cas1)_4_–(Cas2)_2_ structure bound to a splayed DNA duplex [25,26]. The 3′-ssDNA ends are accommodated into two of the four possible Cas1 active sites. DNA ends are bound with sequence specificity because Cas1 recognizes short ‘PAM’ sequences (protospacer adjacent motifs) as part of the DNA capture process, detailed in the next section. The reaction mechanism of integration by *E. coli* Cas1 has been detailed in recent papers [27,28]. Briefly, 3′-OH ends of protospacer DNA bound in Cas1 active sites are used for metal-dependent nucleophilic attack of DNA in a CRISPR locus. The initial integration event occurs at the boundary of the first CRISPR repeat and the leader, and the second site is at the repeat 1–spacer 1 boundary. This covalently attaches the protospacer to CRISPR DNA but generates ssDNA gaps flanking the integration site, which require filling, detailed more below. In *E. coli* Cas1–Cas2 structure, Cas2 acts as a scaffold, to position Cas1 subunits for catalysis; no catalytic activity is required from Cas2 [29]. This topology ensures that *E. coli* Cas1–Cas2 integrates a DNA protospacer of defined size, identified *in vitro* as a 23-bp duplex with 3′ overhangs of five nucleotides in length, providing consistency to the length of spacers integrated into the CRISPR locus. Detailed knowledge of *E. coli* Cas1–Cas2 structure–function belies that there is a significant variation in how Cas1 and its associated proteins achieve adaptation across prokaryotic phyla. Among the type I CRISPR-Cas systems, *Sulfolobus solfataricus* Cas1 does not seem to form a complex with Cas2, yet its reaction mechanism is similar to *E. coli* Cas1 [28], and in *Legionella* species, Cas2 is a DNA/RNA nuclease that links CRISPR immunity with virulence [30]. Other type I systems contain Cas1 proteins fused with Cas4 [31], an iron–sulphur 5′–3′ DNA nuclease [32], indicating that nucleolytic DNA processing during interference and integration are tightly coupled, with or without involvement of Cas2. An ancient CRISPR-Cas precursor ‘Casposon’ from *Aciduliprofundum boonei* has no Cas2 protein, and Cas1 alone is proficient at integrating protospacers into CRISPR as 14–15 bp spacers, much shorter than their counterparts in *E. coli* [33,34]. In type II CRISPR-Cas system of *Streptococcus pyogenes*, which contains the interference enzyme Cas9, a dimer of Cas1 interacts with a tetramer of Csn2 protein [35], suggesting an integration complex that is highly diverged from *E. coli* type I Cas1–Cas2. Cas9 is required for protospacer integration in this system [36], in contrast with *E. coli* in which Cas1–Cas2 alone are sufficient for an integration reaction, albeit one that lacks specificity and would require subsequent gap-filling and DNA ligation, as discussed later.

## Adaptation and interference: priming and capturing of MGE DNA protospacers

The mechanics of protospacer DNA captured from an MGE are less understood than the subsequent protospacer integration, as a new spacer. Protospacer capture can be subdivided into ‘naïve’ and ‘primed’ pathways [37,38]. In naïve adaptation in *E. coli*, spacers from an MGE are generated with absolute dependency on Cas1 and Cas2, but independently of Cascade-catalysed interference. Naïve adaptation could therefore generate immunity against an MGE not previously encountered by the cell. However, the physiological relevance of naïve adaptation in many species may be questionable, especially if, as described later, primed adaptation can also create new spacers even without any escape mutation from an MGE. It is not clear how, in *E. coli*, Cas1–Cas2 would gain access to fragments of protospacer DNA for capture, but the involvement of non-Cas nucleases is a possibility, discussed later.

Primed adaptation manifests as elevated frequencies of spacer incorporation compared with naïve adaptation, in response to previously encountered MGEs [22,39]. In principle this phenomenon, ‘priming’ is the same as observed in metazoan cellular immune systems. Priming is the major driving force for new spacer generation in CRISPR immunity. *E. coli* priming requires Cas1 and Cas2, Cascade and Cas3. Analysis of interplay between these proteins, and perhaps other non-Cas host enzymes, is fascinating in trying to establish how priming occurs during the dynamic operations of the established immune response during an MGE attack, amidst the more routine cellular nucleic acid processing events.

**Figure 2:**
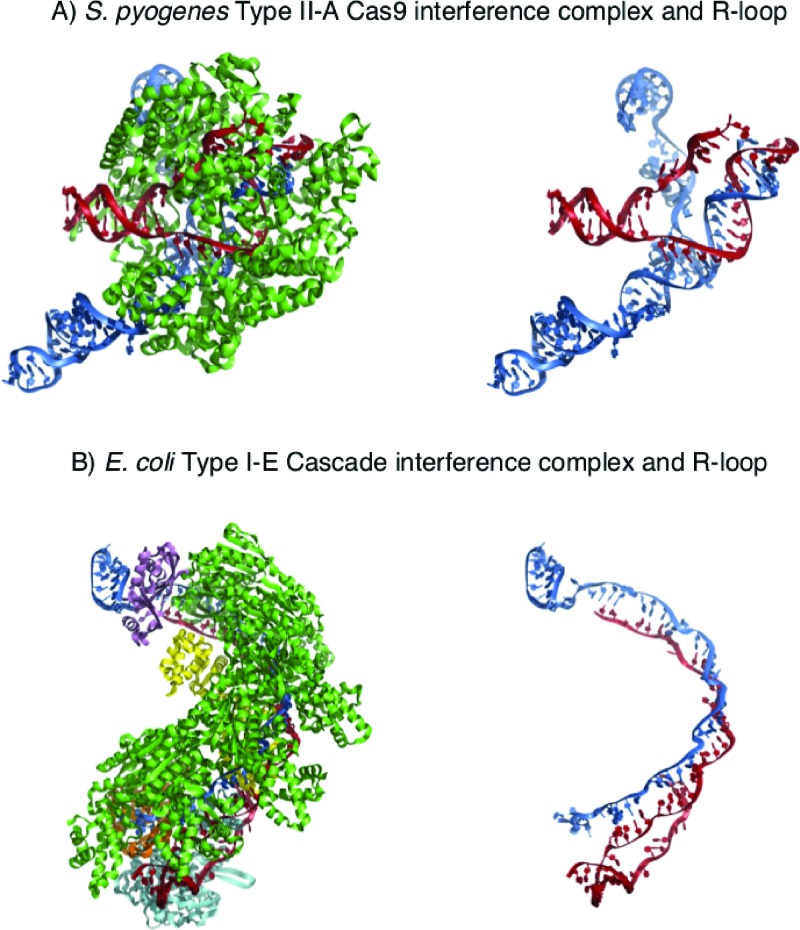
Structural representation of nucleoprotein R-loop complexes. Examples of Cascade and Cas9 interference complexes that form R-loop structures. In both instances crRNA and DNA are coloured as red and blue respectively. (**A**) *S. pyogenes* Cas9 nucleoprotein R-loop forming complex (PBD: 5F9R) shown with/without Cas9 protein (green). (**B**) *E. coli* Cascade nucleoprotein R-loop forming complex (PBD: 5H9E) shown with/without Cascade CasA–E complex (CasA, cyan; CasB, yellow; CasC, green; CasD, orange; CasE, violet).

CRISPR-Cas interference complexes, exemplified by Cascade and Cas9 form nucleoprotein R-loops in which crRNA is base-paired to MGE protospacer DNA (Figure 2). A crucial element for recognition of the protospacer is a trinucleotide ‘PAM’ on MGE DNA, the significance of which was first proposed years before its mechanistic importance was elucidated [40,41]. The nature of PAM recognition dictates that they are directly adjacent to the target site of an interference complex. However variability arises in the exact positioning, with the *E. coli* PAM located on the non-complimentary strand, conversely the *S. pyogenes* PAM is directly upstream of the target sequence on the complimentary strand. Multiple PAMs are often acceptable for Cascades, for example in archaeal organism *Haloferax volcanii* [42], but in *E. coli* there is a preference for the PAM sequence 5′-A[A/T]G-3′ [43]. Recognition of PAM by Cascade subunits, in *E. coli* Cascade by Cse1 (CasA) [44], stimulates R-loop formation and recruits the ATP-dependent Cas3 nuclease-translocase [5,23,45,46], resulting in nicking of non-target strand DNA in the R-loop followed by unidirectional degradation of MGE DNA. In contrast with interference by the Cascade interference complex from *E. coli, S. pyogenes* Cas9 catalyses R-loop formation and nuclease activity via HNH and RuvC-like active sites [47,48]. Cas9 cleaves both strands of the target DNA, generating a DNA double-strand break, and remains DNA-bound before presumably its release by as yet unknown cellular factors. Priming of adaptation is triggered by mutations in PAM, and/or in DNA of the protospacer that result in mismatches with crRNA in interference R-loop [22,49]. Investigating molecular mechanisms of primed adaptation is currently a fast-moving field of research that have highlighted most recently that alterations to Cascade conformation changes its properties, such that Cas3 nuclease may not be directly recruited to a Cascade interference complex, thereby preventing the onset of nuclease stages of interference [50,51]. However, in this scenario Cas1–2 may facilitate recruitment of Cas3, by an unknown mechanism [52]. Bidirectional Cas3 helicase/translocase activity is observed however, leading to the suggestion that *E. coli* Cas3 may transport Cas1–2 along DNA for sampling of MGE DNA for capture [52,53]. Cascade may also recruit Cas3 in a PAM-associated manner, resulting in the cleavage of target DNA into short fragments 30–100 bp in length which can then be processed as new protospacers by/for Cas1–2 to undergo integration [50]. Observations of physical interaction between Cas1–Cas9 in type II-B system, suggests that there is a general principle to facilitate adaptation through physical and functional interaction between proteins of protospacer capture and interference in an ‘adaptasome’ machine. We now look at how non-Cas proteins may contribute to CRISPR-Cas immunity, with emphasis on the roles of proteins hitherto more associated with DNA repair and HR, especially in bacteria.

## Examples of non-Cas host proteins in CRISPR-Cas immunity

**Figure 3:**
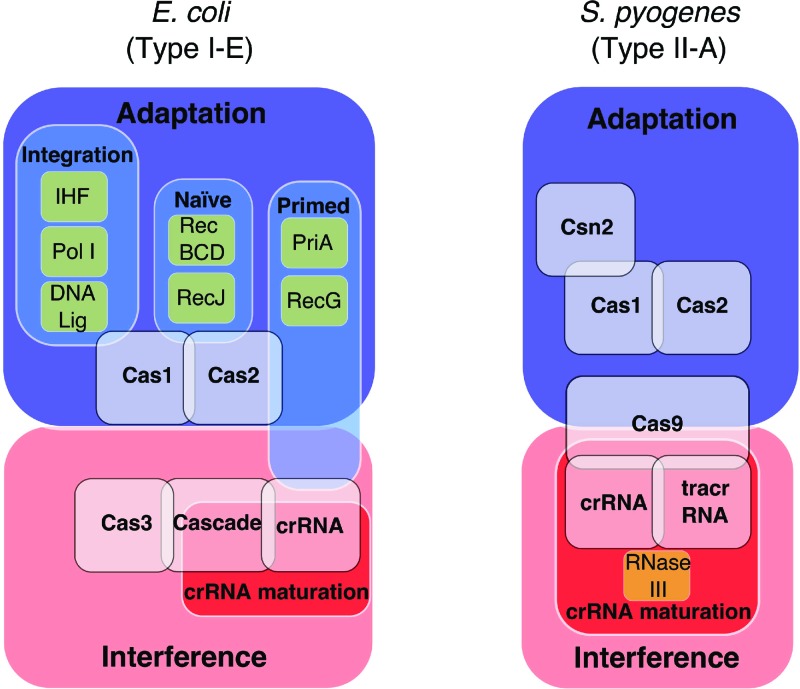
Cas and non-Cas proteins that interplay during CRISPR immunity. Summarised are the Cas and non-Cas proteins that are know to engage in either a physical interaction or functional role during CRISPR immunity in type I-F and type II-A systems of *E. coli* and *S. pyogenes*. Non-Cas *E. coli* proteins are: IHF, Pol I (DNA polymerase I), DNA lig (DNA ligase), RecBCD, RecJ, PriA, and RecG. *E. coli* Cas proteins are; Cas1, Cas2, Cas3 and Cascade (Cas A–E). Non-Cas *S. pyogenes* proteins are: RNase III (Ribonuclease III). *S. pyogenes* Cas proteins: Cas1, Cas2, Cas9, Csn2.

Non-Cas proteins are essential players in promoting, controlling and inhibiting the three stages of CRISPR-Cas immunity (Figure 3). Diversity of CRISPR-Cas systems is mirrored in emerging knowledge of networks for transcriptional regulation of CRISPR-Cas systems in different organisms, reviewed recently [54]. H-NS is a consistent performer across species for regulating CRISPR-Cas systems, acting as a transcriptional repressor, but there are many more unique or specialized effects of other transcriptional regulators. In *E. coli*, the histone-like DNA binding protein IHF (integration host factor) has a crucial role in promoting adaptation in the type I-E CRISPR-Cas system by guiding protospacer bound Cas1–Cas2 to the leading end of a CRISPR locus for integration of a new spacer [55]. *E. coli* IHF was discovered for its ability to promote integration of phage λ, and was subsequently identified as a modulator of recombination, DNA replication and transcription, via sequence-specific manipulation of DNA structures, and by interaction with other nucleic acid processing enzymes [56]. The importance of IHF in CRISPR adaptation, at least in *E. coli*, highlights how fundamental nucleic acid processing mechanisms can be multitasked to underpin more specialized activities. Genetic data from analyses of primed and naïve adaptation in *E. coli* suggest that multiple enzymes of recombination and DNA repair may also support CRISPR adaptation catalysed by Cas1 and Cas2.

## Interactions between CRISPR-Cas and DNA replication, recombination and repair

Experiments in *E. coli* have identified interesting interactions between effectors of HR or DNA repair, and Cas1 or adaptation processes more generally [57-59]. In one analysis [59], *E. coli* cells provoked by genotoxic agents showed reduced viability when the gene encoding Cas1 (*ygbT*) alone was deleted, sensitivity that was epistatic to deletion of genes encoding the HR ‘resolvasome’ proteins RuvABC [60]. This was the first suggestion for potential functional interaction between Cas1 and HR. In the same analysis, purified Cas1 protein physically interacted with RuvB, an ATP-dependent DNA helicase that with RuvA and RuvC branch migrates and resolves Holliday junction DNA molecules formed during late stages of HR [61]. HR can be initiated from dsDNA breaks by resection of the DNA ends by RecBCD protein complex, a bipolar DNA translocase and nuclease [62]. This generates ssDNA that is bound by RecA recombinase and directed into sequence homology searches to generate synaptic products called ‘displacement loops’ (D-loops), that are similar to aforementioned R-loops, but with an invading DNA, not RNA, strand. Interestingly, *E. coli* Cas1 protein also reportedly physically interacts with RecB and RecD [59]. Functional significance of this or of Cas1 interaction with RuvB, is unclear. Genetic analyses of CRISPR adaptation in *E. coli* have identified that RecBCD can be a driver of naïve adaptation [57,58], although it is not needed for primed adaptation [57]. A role for RecBCD in naïve adaptation has been proposed [58], exploiting its ability to recognize a specific DNA sequence on the *E. coli* chromosome called *Chi* (5′-GCTGGTGG-3′). This is proposed to help distinguish ‘self’ from ‘non-self’ DNA, leading to protospacer capture targeted to an MGE rather than the host chromosome. In this model, double-strand breaks in DNA would be necessary, to provide a substrate for canonical RecBCD resection activity. Although these might arise randomly as a result of chemical damage to DNA, it would seem a haphazard way of beginning the CRISPR adaptation process, especially if dsDNA breaks are limited to only (estimated) 10–25 occurrences per cell, per day [63]. It is unclear how the DNA products formed by RecBCD, fragmented ssDNA, even after engagement with *chi*, could become splayed DNA duplexes that are bound by Cas1–Cas2. Some other factor(s) may be able to influence DNA break formation or recombination enzymes may aid and abet CRISPR adaptation via other modes of supporting DNA replication forks [64]. Observation that RecBCD is not needed for primed adaptation [57] suggested that targeting of MGE DNA by Cascade interference, and subsequent Cas3, and/or other, nuclease activity are sufficient for a ‘self-/non-self’ distinction brought about by crRNA of Cascade targeting MGE DNA.

**Figure 4:**
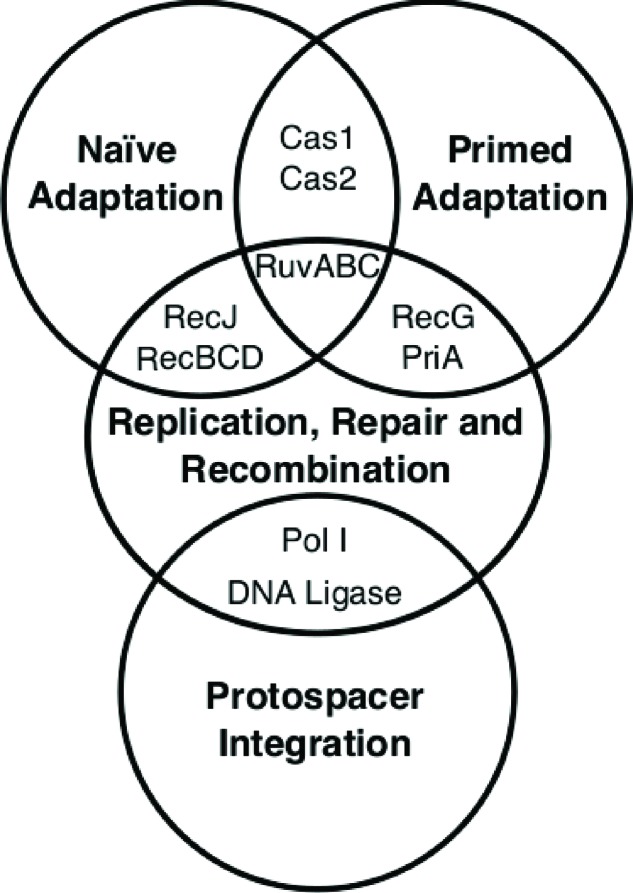
The interplay between the effectors of DNA replication, repair and recombination, and CRISPR-Cas proteins.

In contrast with different requirements for RecBCD in *E. coli* CRISPR, naïve or primed adaptation, a deletion of the gene encoding DNA polymerase I (*polA*) in *E. coli* resulted in no detectable expansion of CRISPR loci in either adaptation pathway [57]. This requirement is compatible with DNA ‘gap filling’, which is required after integration of a new protospacer into a CRISPR locus, and is consistent with the known role of DNA polymerase I in DNA repair pathways. In addition to potential roles for Ruv, RecBCD and DNA polymerase I proteins in adaptation, DNA repair helicases RecG and PriA (sometimes also called primosomal protein N), which are noted for remodelling DNA at blocked replication fork blocks and *ter* sites, have also been shown to be important for primed, but not naïve, adaptation [57]. In one model, it was proposed that Cascade interference complexes might act as roadblocks to MGE replication, which relies on the *E. coli* host replisome. In this model, Cascades form site-specific barriers to MGE replication dictated by the sequence of crRNA, and that this is a trigger point for adaptation by interaction with Cas1–Cas2. Roles for RecG and PriA in this model are unclear, although we speculate that the known ability of RecG to dissociate R-loops may be important for controlling access of Cas enzymes to DNA and potentially to prevent R-loop primed DNA replication, which is a known cause of genome stability. Overall physical and function interplay between effectors of DNA replication, repair and recombination, and CRISPR-Cas proteins (summarized in Figure 4) are significant but poorly understood, requiring further work to establish mechanism and especially if CRISPR interference antagonizes DNA replication.

## Abbreviations

Cas: CRISPR-associated protein
CRISPR: Clustered Regularly Interspersed Short Palindromic Repeats
crRNA: CRISPR RNA
HR: homologous recombination
IHF: integration host factor
MGE: mobile genetic element
PAM: protospacer adjacent motif
R-loop: RNA-loop

## References

[1] Mohanraju P., Makarova K.S., Zetsche B., Zhang F., Koonin E.V. and van der Oost J. (2016) Diverse evolutionary roots and mechanistic variations of the CRISPR-Cas systems. Science 353, aad5147 27493190 10.1126/science.aad5147 PMC13189112

[2] Jackson S.A., McKenzie R.E., Fagerlund R.D., Kieper S.N., Fineran P.C. and Brouns S.J. (2017) CRISPR-Cas: adapting to change. Science 356 10.1126/science.aal5056 28385959

[3] Makarova K.S., Wolf Y.I. and Koonin E.V. (2013) The basic building blocks and evolution of CRISPR-CAS systems. Biochem. Soc. Trans. 41, 1392–1400 24256226 10.1042/BST20130038 PMC5898231

[4] Makarova K.S., Wolf Y.I., Alkhnbashi O.S., Costa F., Shah S.A., Saunders S.J. (2015) An updated evolutionary classification of CRISPR-Cas systems. Nat. Rev. Microbiol. 13, 722–736 26411297 10.1038/nrmicro3569 PMC5426118

[5] Brouns S.J., Jore M.M., Lundgren M., Westra E.R., Slijkhuis R.J., Snijders A.P. (2008) Small CRISPR RNAs guide antiviral defense in prokaryotes. Science 321, 960–964 18703739 10.1126/science.1159689 PMC5898235

[6] Makarova K.S., Zhang F. and Koonin E.V. (2017) SnapShot: class 1 CRISPR-Cas systems. Cell 168, 946–946.e1 28235204 10.1016/j.cell.2017.02.018

[7] Makarova K.S., Zhang F. and Koonin E.V. (2017) SnapShot: class 2 CRISPR-Cas systems. Cell 168, 328–328.e1 28086097 10.1016/j.cell.2016.12.038

[8] Barrangou R. (2015) The roles of CRISPR-Cas systems in adaptive immunity and beyond. Curr. Opin. Immunol. 32C, 36–41 10.1016/j.coi.2014.12.008 25574773

[9] Mojica F.J. and Montoliu L. (2016) On the origin of CRISPR-Cas technology: from prokaryotes to mammals. Trends Microbiol. 24, 811–820 27401123 10.1016/j.tim.2016.06.005

[10] Mojica F.J. and Rodriguez-Valera F. (2016) The discovery of CRISPR in archaea and bacteria. FEBS J. 283, 3162–3169 27234458 10.1111/febs.13766

[11] Mojica F.J. (2016) An interview with Francisco Mojica. Lab. Times 6, 16–20

[12] Doudna J.A. and Charpentier E. (2014) Genome editing. The new frontier of genome engineering with CRISPR-Cas9. Science 346, 1258096 25430774 10.1126/science.1258096

[13] Dong D., Ren K., Qiu X., Zheng J., Guo M., Guan X. (2016) The crystal structure of Cpf1 in complex with CRISPR RNA. Nature 532, 522–526 27096363 10.1038/nature17944

[14] Fonfara I., Richter H., Bratovic M., Le Rhun A. and Charpentier E. (2016) The CRISPR-associated DNA-cleaving enzyme Cpf1 also processes precursor CRISPR RNA. Nature 532, 517–521 27096362 10.1038/nature17945

[15] Yamano T., Nishimasu H., Zetsche B., Hirano H., Slaymaker I.M., Li Y. (2016) Crystal structure of Cpf1 in complex with guide RNA and target DNA. Cell 165, 949–962 27114038 10.1016/j.cell.2016.04.003 PMC4899970

[16] White M.F. (2016) Cpf1 shape-shifts for streamlined CRISPR cleavage. Nat. Struct. Mol. Biol. 23, 365–366 27142322 10.1038/nsmb.3225

[17] Zetsche B., Gootenberg J.S., Abudayyeh O.O., Slaymaker I.M., Makarova K.S., Essletzbichler P. (2015) Cpf1 is a single RNA-guided endonuclease of a class 2 CRISPR-Cas system. Cell 163, 759–771 26422227 10.1016/j.cell.2015.09.038 PMC4638220

[18] Yang H., Gao P., Rajashankar K.R. and Patel D.J. (2016) PAM-dependent target dna recognition and cleavage by C2c1 CRISPR-Cas endonuclease. Cell 167, 1814–1828.e12 27984729 10.1016/j.cell.2016.11.053 PMC5278635

[19] Thakore P.I., Black J.B., Hilton I.B. and Gersbach C.A. (2016) Editing the epigenome: technologies for programmable transcription and epigenetic modulation. Nat. Methods 13, 127–137 26820547 10.1038/nmeth.3733 PMC4922638

[20] Chaikind B., Bessen J.L., Thompson D.B., Hu J.H. and Liu D.R. (2016) A programmable Cas9-serine recombinase fusion protein that operates on DNA sequences in mammalian cells. Nucleic Acids Res. 44, 9758–9770 27515511 10.1093/nar/gkw707 PMC5175349

[21] Guilinger J.P., Thompson D.B. and Liu D.R. (2014) Fusion of catalytically inactive Cas9 to FokI nuclease improves the specificity of genome modification. Nat. Biotechnol. 32, 577–582 24770324 10.1038/nbt.2909 PMC4263420

[22] Datsenko K.A., Pougach K., Tikhonov A., Wanner B.L., Severinov K. and Semenova E. (2012) Molecular memory of prior infections activates the CRISPR/Cas adaptive bacterial immunity system. Nat. Commun. 3, 945 22781758 10.1038/ncomms1937

[23] Jore M.M., Lundgren M., van Duijn E., Bultema J.B., Westra E.R., Waghmare S.P. (2012) Structural basis for CRISPR RNA-guided DNA recognition by Cascade. Nat. Struct. Mol. Biol. 18, 529–536 10.1038/nsmb.2019 21460843

[24] Szczelkun M.D., Tikhomirova M.S., Sinkunas T., Gasiunas G., Karvelis T., Pschera P. (2014) Direct observation of R-loop formation by single RNA-guided Cas9 and Cascade effector complexes. Proc. Natl. Acad. Sci. U.S.A 111, 9798–9803 24912165 10.1073/pnas.1402597111 PMC4103346

[25] Wang J., Li J., Zhao H., Sheng G., Wang M., Yin M. (2015) Structural and mechanistic basis of PAM-dependent spacer acquisition in CRISPR-Cas systems. Cell 163, 840–853 26478180 10.1016/j.cell.2015.10.008

[26] Nunez J.K., Harrington L.B., Kranzusch P.J., Engelman A.N. and Doudna J.A. (2015) Foreign DNA capture during CRISPR-Cas adaptive immunity. Nature 527, 535–538 26503043 10.1038/nature15760 PMC4662619

[27] Nunez J.K., Lee A.S., Engelman A. and Doudna J.A. (2015) Integrase-mediated spacer acquisition during CRISPR-Cas adaptive immunity. Nature 519, 193–198 25707795 10.1038/nature14237 PMC4359072

[28] Rollie C., Schneider S., Brinkmann A.S., Bolt E.L. and White M.F. (2015) Intrinsic sequence specificity of the Cas1 integrase directs new spacer acquisition. Elife 4, DOI 10.7554/eLife.08716 10.7554/eLife.08716 PMC4574026 26284603

[29] Nunez J.K., Kranzusch P.J., Noeske J., Wright A.V., Davies C.W. and Doudna J.A. (2014) Cas1-Cas2 complex formation mediates spacer acquisition during CRISPR-Cas adaptive immunity. Nature Struct. Mol. Biol. 21, 528–534 24793649 10.1038/nsmb.2820 PMC4075942

[30] Gunderson F.F., Mallama C.A., Fairbairn S.G. and Cianciotto N.P. (2015) Nuclease activity of *Legionella pneumophila* Cas2 promotes intracellular infection of amoebal host cells. Infect. Immun. 83, 1008–1018 25547789 10.1128/IAI.03102-14 PMC4333442

[31] Viswanathan P., Murphy K., Julien B., Garza A.G. and Kroos L. (2007) Regulation of dev, an operon that includes genes essential for *Myxococcus xanthus* development and CRISPR-associated genes and repeats. J. Bacteriol. 189, 3738–3750 17369305 10.1128/JB.00187-07 PMC1913320

[32] Zhang J., Kasciukovic T. and White M.F. (2012) The CRISPR associated protein Cas4 is a 5′ to 3′ DNA exonuclease with an iron-sulfur cluster. PLoS ONE 7, e47232 23056615 10.1371/journal.pone.0047232 PMC3466216

[33] Hickman A.B. and Dyda F. (2015) The casposon-encoded Cas1 protein from* Aciduliprofundum boonei* is a DNA integrase that generates target site duplications. Nucleic Acids Res. 43, 10576–10587 26573596 10.1093/nar/gkv1180 PMC4678821

[34] Krupovic M., Makarova K.S., Forterre P., Prangishvili D. and Koonin E.V. (2014) Casposons: a new superfamily of self-synthesizing DNA transposons at the origin of prokaryotic CRISPR-Cas immunity. BMC Biol. 12, 36 24884953 10.1186/1741-7007-12-36 PMC4046053

[35] Ka D., Lee H., Jung Y.D., Kim K., Seok C., Suh N. (2016) Crystal structure of *Streptococcus pyogenes* Cas1 and its interaction with Csn2 in the type II CRISPR-Cas system. Structure 24, 70–79 26671707 10.1016/j.str.2015.10.019

[36] Heler R., Samai P., Modell J.W., Weiner C., Goldberg G.W., Bikard D. (2015) Cas9 specifies functional viral targets during CRISPR-Cas adaptation. Nature 519, 199–202 25707807 10.1038/nature14245 PMC4385744

[37] Fineran P.C. and Charpentier E. (2012) Memory of viral infections by CRISPR-Cas adaptive immune systems: acquisition of new information. Virology 434, 202–209 23123013 10.1016/j.virol.2012.10.003

[38] Yosef I., Goren M.G. and Qimron U. (2012) Proteins and DNA elements essential for the CRISPR adaptation process in *Escherichia coli*. Nucleic Acids Res. 40, 5569–5576 22402487 10.1093/nar/gks216 PMC3384332

[39] Fineran P.C., Gerritzen M.J., Suarez-Diez M., Kunne T., Boekhorst J., van Hijum S.A. (2014) Degenerate target sites mediate rapid primed CRISPR adaptation. Proc. Natl. Acad. Sci. U.S.A. 111, E1629–E1638 24711427 10.1073/pnas.1400071111 PMC4000823

[40] Bolotin A., Quinquis B., Sorokin A. and Ehrlich S.D. (2005) Clustered regularly interspaced short palindrome repeats (CRISPRs) have spacers of extrachromosomal origin. Microbiology 151, 2551–2561 16079334 10.1099/mic.0.28048-0

[41] Mojica F.J., Diez-Villasenor C., Garcia-Martinez J. and Almendros C. (2009) Short motif sequences determine the targets of the prokaryotic CRISPR defence system. Microbiology 155, 733–740 19246744 10.1099/mic.0.023960-0

[42] Fischer S., Maier L.K., Stoll B., Brendel J., Fischer E., Pfeiffer F. (2012) An archaeal immune system can detect multiple protospacer adjacent motifs (PAMs) to target invader DNA. J. Biol. Chem. 287, 33351–33363 22767603 10.1074/jbc.M112.377002 PMC3460438

[43] Westra E.R., Semenova E., Datsenko K.A., Jackson R.N., Wiedenheft B., Severinov K. (2013) Type I-E CRISPR-cas systems discriminate target from non-target DNA through base pairing-independent PAM recognition. PLoS Genet. 9, e1003742 24039596 10.1371/journal.pgen.1003742 PMC3764190

[44] Sashital D.G., Wiedenheft B. and Doudna J.A. (2012) Mechanism of foreign DNA selection in a bacterial adaptive immune system. Mol. Cell 46, 606–615 22521690 10.1016/j.molcel.2012.03.020 PMC3397241

[45] Hochstrasser M.L., Taylor D.W., Bhat P., Guegler C.K., Sternberg S.H., Nogales E. (2014) CasA mediates Cas3-catalyzed target degradation during CRISPR RNA-guided interference. Proc. Natl. Acad. Sci. U.S.A. 111, 6618–6623 24748111 10.1073/pnas.1405079111 PMC4020112

[46] Wiedenheft B., Lander G.C., Zhou K., Jore M.M., Brouns S.J., van der Oost J. (2011) Structures of the RNA-guided surveillance complex from a bacterial immune system. Nature 477, 486–489 21938068 10.1038/nature10402 PMC4165517

[47] Jinek M., Jiang F., Taylor D.W., Sternberg S.H., Kaya E., Ma E. (2014) Structures of Cas9 endonucleases reveal RNA-mediated conformational activation. Science 343, 1247997 24505130 10.1126/science.1247997 PMC4184034

[48] Jinek M., Chylinski K., Fonfara I., Hauer M., Doudna J.A. and Charpentier E. (2012) A programmable dual-RNA-guided DNA endonuclease in adaptive bacterial immunity. Science 337, 816–821 22745249 10.1126/science.1225829 PMC6286148

[49] Wiedenheft B., van Duijn E., Bultema J.B., Waghmare S.P., Zhou K., Barendregt A. (2011) RNA-guided complex from a bacterial immune system enhances target recognition through seed sequence interactions. Proc. Natl. Acad. Sci. U.S.A. 108, 10092–10097 21536913 10.1073/pnas.1102716108 PMC3121849

[50] Kunne T., Kieper S.N., Bannenberg J.W., Vogel A.I., Miellet W.R., Klein M. (2016) Cas3-derived target dna degradation fragments fuel primed CRISPR adaptation. Mol. Cell 63, 852–864 27546790 10.1016/j.molcel.2016.07.011

[51] Blosser T.R., Loeff L., Westra E.R., Vlot M., Kunne T., Sobota M. (2015) Two distinct DNA binding modes guide dual roles of a CRISPR-Cas protein complex. Mol. Cell 58, 60–70 25752578 10.1016/j.molcel.2015.01.028 PMC4475636

[52] Redding S., Sternberg S.H., Marshall M., Gibb B., Bhat P., Guegler C.K. (2015) Surveillance and processing of foreign DNA by the *Escherichia coli* CRISPR-Cas system. Cell 163, 854–865 26522594 10.1016/j.cell.2015.10.003 PMC4636941

[53] Sternberg S.H., Redding S., Jinek M., Greene E.C. and Doudna J.A. (2014) DNA interrogation by the CRISPR RNA-guided endonuclease Cas9. Nature 507, 62–67 24476820 10.1038/nature13011 PMC4106473

[54] Patterson A.G., Yevstigneyeva M.S. and Fineran P.C. (2017) Regulation of CRISPR-Cas adaptive immune systems. Curr. Opin. Microbiol. 37, 1–7 28359988 10.1016/j.mib.2017.02.004

[55] Nunez J.K., Bai L., Harrington L.B., Hinder T.L. and Doudna J.A. (2016) CRISPR immunological memory requires a host factor for specificity. Mol. Cell 62, 824–833 27211867 10.1016/j.molcel.2016.04.027

[56] Travers A. (1997) DNA-protein interactions: IHF–the master bender. Curr. Biol. 7, R252–R254 9162504 10.1016/s0960-9822(06)00114-x

[57] Ivancic-Bace I., Cass S.D., Wearne S.J. and Bolt E.L. (2015) Different genome stability proteins underpin primed and naive adaptation in E. coli CRISPR-Cas immunity. Nucleic Acids Res. 43, 10821–10830 26578567 10.1093/nar/gkv1213 PMC4678826

[58] Levy A., Goren M.G., Yosef I., Auster O., Manor M., Amitai G. (2015) CRISPR adaptation biases explain preference for acquisition of foreign DNA. Nature 520, 505–510 25874675 10.1038/nature14302 PMC4561520

[59] Babu M., Beloglazova N., Flick R., Graham C., Skarina T., Nocek B. (2011) A dual function of the CRISPR-Cas system in bacterial antivirus immunity and DNA repair. Mol. Microbiol. 79, 484–502 21219465 10.1111/j.1365-2958.2010.07465.x PMC3071548

[60] Whitby M.C., Bolt E.L., Chan S.N. and Lloyd R.G. (1996) Interactions between RuvA and RuvC at Holliday junctions: inhibition of junction cleavage and formation of a RuvA-RuvC-DNA complex. J. Mol. Biol. 264, 878–890 9000618 10.1006/jmbi.1996.0684

[61] Whitby M.C., Sharples G.J. and Lloyd R.G. (1995) The RuvAB and RecG proteins of *Escherichia coli*. Nucleic Acids Mol. Biol. 9, 66–83

[62] Singleton M.R., Dillingham M.S., Gaudier M., Kowalczykowski S.C. and Wigley D.B. (2004) Crystal structure of RecBCD enzyme reveals a machine for processing DNA breaks. Nature 432, 187–193 15538360 10.1038/nature02988

[63] Tubbs A. and Nussenzweig A. (2017) Endogenous DNA damage as a source of genomic instability in cancer. Cell 168, 644–656 28187286 10.1016/j.cell.2017.01.002 PMC6591730

[64] Cox M.M., Goodman M.F., Kreuzer K.N., Sherratt D.J., Sandler S.J. and Marians K.J. (2000) The importance of repairing stalled replication forks. Nature 404, 37–41 10716434 10.1038/35003501

